# Coping strategies mediate the association between stigma and fertility quality of life in infertile women undergoing in vitro fertilization-embryo transfer

**DOI:** 10.1186/s12905-021-01525-9

**Published:** 2021-11-02

**Authors:** Xiaoyu Jing, Wei Gu, Lu Zhang, Runna Miao, Xiuli Xu, Min Wang, Hadassah Joann Ramachandran, Wenru Wang

**Affiliations:** 1 School of Nursing, Xi’an Jiaotong University Health Science Centre, No. 76 Yantaxi Road, Xi’an, 710061 Shaanxi China; 2 Northwest Women’s and Children’s Hospital, No. 73 Houzaimen, Xi’an, 710003 Shaanxi China; 3 Alice Lee Centre for Nursing Studies, Yong Loo Lin School of Medicine, National University of Singapore, Block MD11, level 2 10 Medical Drive, Singapore, 117597 Singapore

**Keywords:** Infertility, Stigma, Coping strategy, Fertility quality of life, Moderating role

## Abstract

**Background:**

The mediating role of coping strategies and its relationship with psychological well-being and quality of life has been considered in the literature. However, there is little research to explore the mechanism of coping strategies on stigma and fertility quality of life (FertiQoL) in infertile women undergoing In Vitro Fertilization Embryo Transfer (IVF-ET). The aim of this study was to examine the mediating effect of coping strategies on the relationship between stigma and fertility quality of life (FertiQoL) in Chinese infertile women undergoing In Vitro Fertilization Embryo Transfer (IVF-ET).

**Methods:**

In this cross-sectional study, a total of 768 infertile women undergoing IVF-ET were recruited from Assisted Reproductive Center of Shaanxi Province, China. The personal information, infertility stigma scale, coping strategy scale and FertiQoL scale were measured using a set of questionnaires. The multiple mediator model was performed using AMOS 21.0.

**Results:**

The model showed a significant negative direct effect between stigma on FertiQoL. There were significantly negative indirect effects of stigma on FertiQoL through active-avoidance, active-confronting and passive-avoidance, respectively. The meaning-based coping played a positive intermediary role. The model explained 69.4% of the variance in FertiQoL.

**Conclusion:**

Active-avoidance coping strategy is the most important mediator factor between stigma and FertiQoL in infertile women undergoing IVF-ET treatment. Meaning-based coping strategy plays a positive mediating role between stigma and FertiQoL.

## Introduction

Infertility—characterized by the inability of an individual or couple to conceive or have a successful pregnancy after 12 months of regular, unprotected sexual intercourse—is a prevalent disease that affects over 186 million people worldwide [1, 2]. Statistical figures have indicated that infertility increases with age, and a recent study in China found that the prevalence of infertility among women of childbearing age was 15.5% [3]. Since the 1970s, In Vitro Fertilization Embryo Transfer (IVF-ET) has seen steady increases in utilization and remains a hope for many infertile women. However, pregnancies via IVF-ET are vulnerable to greater risks of miscarriage compared to spontaneous pregnancies, and uncertain pregnancy outcomes may bring infertile women serious psychological burden [4, 5], such as anxiety, depression and stigma, which has been shown to seriously affect their FertiQoL [6, 7].

FertiQoL is an individual’s perception and satisfaction with all aspects of life when faced with fertility problems with better FertiQoL of infertile women during infertility treatment being an important outcome indicator in the new medical model [8]. However, studies have convincingly demonstrated that when compared with their fertile counterparts, women undergoing infertility treatment experienced poorer FertiQoL. Numerous factors such as higher level of education, infertility-related stress and stigma has been shown to lower FertiQoL [9–11]. Specifically, stigma was found to be a strong predictor of FertiQoL among Chinese infertile women undergoing IVF-ET [12].

Stigma is a negative psychological attitude, which has been linked with an array of negative consequences. In many societies, including China, the stigma created by infertility and consequent childlessness is based on a deviation from the social norm to procreate, and often leaves individuals with feelings of guilt in managing both their infertility and stigmatization [13]. The stigmatization of women who suffer from infertility and humiliation from family members and the public eye has effects on self-devaluation or social withdrawal. As a result, infertile women are left with a strong sense of loneliness, social and emotional stress, and poor social relationships [14]. Researches showed that women undergoing infertility treatments bear a heavy family and public stigma and a lower quality of life [12, 15, 16]. However, there is no research to explore the how stigma affects FertiQoL of infertile women who undergoing IVF-ET treatment.

The mediating role of coping strategies and its relationship with psychological well-being and quality of life has been considered in the literature [17–19]. According to Schmidt’s et al., the coping strategies adopted by women facing infertility-pressure can be classified into four categories: active-avoidance, active-confronting, passive-avoidance and meaning-based coping [20]. Active-confronting coping strategies like actively seeking medical advice or seeking help can reduce the negative impact of stigma on the quality of life [21, 22],whereas avoidance coping strategy has been linked to self-devaluation, social withdrawal and declining FertiQoL in infertile women [23]. However, there is little research to explore the pathway of coping strategies on stigma and FertiQoL.

The aim of the study was to examine the mediating effect of coping strategies on the relationship between stigma and FertiQoL in Chinese infertile women undergoing IVF-ET. We hypothesized that (a) there is a negative correlation between stigma and FertiQoL in infertile Chinese women undergoing IVF-ET treatment; and (b) the four coping strategies play a mediating role between stigma and FertiQoL in infertile women undergoing IVF-ET treatment.

## Methods

### Design and participants

This study is part of the Research Program of FertiQoL in Infertile Women which is a cross-sectional evaluation of FertiQoL among infertile women in China. A convenience sampling method was used to recruit infertile women from the Assisted Reproductive Center of Shaanxi Province, China from September 2018 to November 2019. The inclusion criteria included women who (a) were diagnosed with infertility and undergoing IVF-ET treatment; (b) aged between 20 and 45 years; and (c) were able to communicate in Chinese. Women with mental disorders or cognitive impairment were excluded.

Multiple linear regression analysis was used to identify the predictive factors of FertiQoL of infertile women undergoing IVF-ET treatment. Based on literature reviews, we had selected a total of 32 factors as independent variables (i.e. educational level, residence, duration of infertility, stigma, and coping strategy as independent variables), the sample size was 10–20 times of the independent variables. We considered a 20% loss rate, and according to the minimum sample size requirement (> 200) of the structural equation [24]. The final sample size reached for this study was 768.

### Data collection

The data of 768 infertile women undergoing IVF-ET were collected from September 2018 to November 2019. The researcher approached potential participants in the waiting area of the IVF-ET operating room on the day of embryo transfer at the study center. Eligible participants were selected based on the inclusion criteria. A nurse employed by the study center assisted the researcher to approach potential participants. The four self-reported scales were administered online using QR scan. A total of 800 infertile women attempted the questionnaire and each questionnaire took between 20 and 30 min to complete. 32 questionnaires had missing or incorrect data and were excluded from the study (response rate = 96%).

### Measures

#### The personal information questionnaire

Information on personal demographics and medical background including age, educational level, residence, occupation, duration of infertility, infertility type, and cycles of IVF-ET were collected.

#### Infertility Stigma Scale (ISS)

The ISS, developed by Fu et al. (2015), is a 27-item scale with four subscales and is considered to be a measure of women's perceived stigma and self-stigma in the diagnosis and treatment of infertility [25]. The perceived self-stigma of infertile women were assessed in self-devaluation (7 items), social withdrawal (5items), public stigma (9 items) and family stigma (6 items). All items are rated on a 5-point Likert scale of 1 (totally disagree) to 5 (totally agree). Total score ranges from 27 to 135, with higher scores indicating increased levels of stigma. The ISS has been demonstrated to have good reliability, with Cronbach's alpha coefficient of 0.94. The Cronbach's alpha coefficient was 0.95 in this study.

#### Coping Strategy Scale

The coping strategy scale used was Schmidt’s et al. version of Copenhagen Multicenter Psychosocial infertility (COMPI) Coping Strategy Scale [20]. The 19-item scale assesses how often infertile women engaged in various coping strategies in response to a particular fertility pressure. The coping strategy scale is categorized into four subscales: (1) active-avoidance strategy (e.g., avoiding pregnant women); (2) active-confronting strategy (e.g., asking medical workers for help); (3) passive-avoidance strategy (e.g., looking forward to miracles); (4) meaning-based coping strategy (e.g., finding other goals in life from infertility). Cronbach’s alpha of the four original subscales of the Coping Strategy Scale was 0.68, 0.76, 0.46 and 0.59, respectively. In this study, the Cronbach’s alpha ranged from 0.60 to 0.74 for the subscales.

#### Fertility Quality of Life Scale (FertiQoL scale)

The FertiQoL scale was used to assess the quality of life of the participants [8]. The FertiQoL scale consists of 36 items, out of which 2 single items are used to assess general health and life satisfaction. The remaining 34 items are comprised of two domains: Core FertiQoL and Treatment FertiQoL. Core FertiQoL consists of four subscales: emotional (6 items), mind–body (6 items), relational (6 items) and social (6 items). Treatment FertiQoL consists of two subscales: treatment environment (6 items) and treatment tolerability (4 items). This is a 5-point Likert scale with total scores ranging from 0 to 100. The Chinese version of the FertiQoL has also demonstrated good validity and reliability with Cronbach's alpha of 0.925. The Cronbach's alpha coefficient of FertiQoL in this study was 0.906.

### Ethics considerations

Ethics approval was obtained from the study hospital (Ref. No. 2019.015). All study participants were guaranteed confidentiality and were informed that their participation was voluntary.

### Data analysis

The SPSS24.0 and AMOS21.0 were used to analyze the data. Pearson’s correlation was used to test the correlations between ISS, coping strategy and FertiQoL scores. To determine the mediating role of coping strategies, multiple mediator model was performed for each independent variable separately. Four mediators (active-avoidance, active-confronting, passive-avoidance, meaning-based coping) were tested. The significance of indirect effect was tested using percentile bootstrapping to estimate the standard error and the 95% confidence interval.

## Result

### Characteristics of infertile women

The characteristics of the participants are presented in Table 1. A total of 768 infertile women undergoing IVF-ET completed the online questionnaire survey. The mean age of the participants was 30.93 (SD = 4.08). About half of infertile women (n = 414, 53.9%) had secondary infertility, however majority (n = 667, 86.8%) were undergoing their first IVF-ET (Table 1).

**Table 1:** Characteristics of infertile women undergoing IVF-ET treatment

Item	n	%
*Age*		
20–25	53	6.9
26–31	416	54.2
32–37	238	31.0
> 37	61	7.9
*Educational level*		
Primary	183	23.8
Secondary	168	21.9
University	417	54.3
*Residence*		
Countryside	260	33.9
Town	166	21.6
City	342	44.5
*Length of marriage (year)*		
1–3	343	44.7
4–6	247	32.2
> 6	178	23.1
*Financial condition (Chinese ¥)*		
≤ 3000	295	38.4
3001–5000	320	41.7
> 5000	153	19.9
*Duration of infertility (year)*		
≤ 3	474	61.7
> 3	294	38.3
*Religious beliefs*		
Yes	20	2.6
No	748	97.4
*Type of infertility*		
Primary infertility	354	46.1
Secondary infertility	414	53.9
*History of infertility treatment*		
Yes	322	41.9
No	446	58.1
*History of IVF-ET treatment*		
Yes	101	13.2
No	667	86.8
*Insurance*		
Yes	276	35.9
No	492	64.1

### Correlation among subscales of ISS, coping strategy and FertiQoL

There were significant negative correlations between four coping strategies and FertiQoL (r = − 0.124–0.611, p < 0.001). A significantly positive correlation between stigma and four coping strategies was found (r = 0.157–0.592, p < 0.001) (Table 2).

**Table 2:** Means, standard deviation and Pearson correlations (Pearson r) among study variables

Variables	Mean	SD	1	2	3	4	5	6
1. Stigma	61.64	20.62	1					
2. Active-avoidance	8.67	2.86	0.592***	1				
3. Active-confronting	14.85	4.13	0.381***	0.497***	1			
4. Passive-avoidance	8.34	2.32	0.375***	0.531***	0.444***	1		
5. Meaning-based	12.98	3.27	0.157***	0.399***	0.535***	0.380***	1	
6. FertiQoL	63.94	13.09	− 0.686***	− 0.611***	− 0.409***	− 0.406***	− 0.124***	1

1: stigma; 2: Active-avoidance; 3: Active-confronting; 4: Passive-avoidance; 5: Meaning-based; 6: FertiQoL

^***^p < 0.01

### The mediating roles of coping strategies

**Fig. 1:**
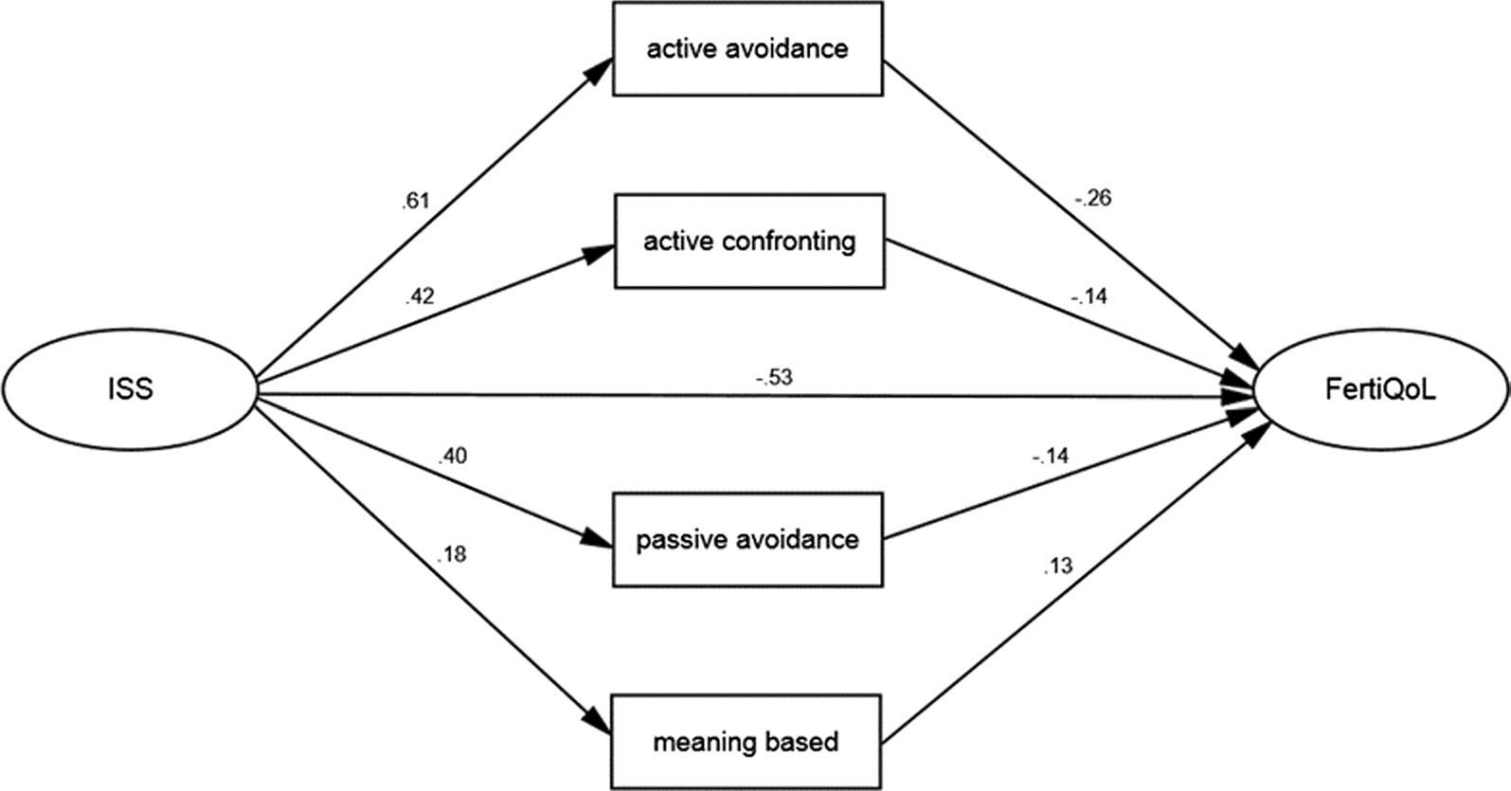
Coping strategies as mediators in the relationship between stigma and FertiQoL

Figure 1 shows the mediation model with standardized regression coefficients. Indices of goodness-of-fit of the model showed a desirable fit, with CMIN/DF = 3.213, GFI = 0.970, CFI = 0.982 and RMSEA = 0.054. The model showed a significant negative direct effect between stigma and FertiQoL (direct effect = − 0.53, p < 0.001). There were also significantly negative indirect effects of stigma on FertiQoL through active-avoidance (indirect effect = − 0.16, p < 0.001), active-confronting (indirect effect = − 0.06, p < 0.001) and passive-avoidance (indirect effect = − 0.06, p < 0.001) respectively. Although there was a significantly positive mediating effect of meaning-based between stigma and FertiQoL (indirect effect = 0.02, p < 0.001), the total effect was negative (total effect = − 0.79, p < 0.001). The total squared multiple correlation between stigma and FertiQoL was 0.694, it explained 69.4% of the variance in FertiQoL (Fig. 1 and Table 3).

**Table 3:** Standardized direct, indirect, and total effects for hypothetical model (N = 768)

Path	Direct effect 1	Direct effect 2	Indirect effect	Total effect	SMC (R^2^)
Stigma → Active-avoidance → FertiQoL	0.61^***^	− 0.26^***^	− 0.16^***^	− 0.69^***^	0.374
Stigma → Active-confronting → FertiQoL	0.42^***^	− 0.14^***^	− 0.06^***^	− 0.59^***^	0.179
Stigma → Passive avoidance → FertiQoL	0.40^***^	− 0.14^***^	− 0.06^***^	− 0.59^***^	0.160
Stigma → Meaning-based → FertiQoL	0.18^***^	0.13^***^	0.02^***^	− 0.51^***^	0.031
Stigma → FertiQoL	− 0.53^***^		− 0.26^***^	− 0.79***	0.694

^***^p < 0.001

## Discussion

Our study results showed that avoidance coping strategy played the strongest stigma on FertiQoL. This has also been demonstrated in previous studies where stigma was positively correlated with avoidance coping strategy, while avoidance coping strategy was negatively related to FertiQoL [26, 27]. This indicates that infertile women with high levels of stigma were more likely to avoid contact with pregnant women or children, especially during infertility treatment. This inadvertently reduces their social communication and interaction and leads to a deterioration of their FertiQoL. In infertile women, avoidance strategies indicate a lack of confidence in the effectiveness of treatment. Although avoidance strategies can temporarily distract and relieve the mental pressures, this is often not sustainable over long in-vitro transplantation treatment periods, and may further aggravate helplessness and stigma, resulting in decreased FertiQoL. Our result is also consistent with studies conducted on other patient populations in China—convalescent schizophrenia [28, 29] and pulmonary cancer in—where the more patients used avoidance coping strategy, the greater the negative effect of stigma on quality of life. This similarity in the role of avoidance coping across different patient populations with different dimensions of stigma and QoL has wider implications on the importance of considering coping mechanisms as part of holistic clinical care and patient education.

The multiple mediator model (Fig. 1) showed that active-confronting coping strategy also played a negatively moderating role between stigma and FertiQoL. This is not in line with other studies, which reported that positive active coping strategies could reduce the negative impact of stigma on quality of life [21]. Folkman et al proposed that the effectiveness of a particular coping strategy is dependent on the match or goodness of fit between the strategy and the controllability of the event [30]. Considering that undergoing IVF-ET treatment is an extremely complicated and uncontrollable treatment where results are often uncertain, the use of an active medical seeking behavior may have proved counter-productive to the 86.8% of infertile women in this study. Efforts of infertile women to manage their treatment actively may engender feelings of frustration and disappointment, which is likely to have deleterious effects on infertile women themselves. This is echoed by Terry et al., who reported that the effect of active coping strategies was variable when an individual faced an uncontrollable stressor [31]. Conversely, our results showed that meaning-based coping strategy played a positive mediating role between stigma and FertiQoL. Infertile women who respond to infertility by praying or finding other goals can achieve better FertiQoL and lower stigma. This may lie in the fact that the meaning-based coping strategy can transfer the infertility pressure and make infertile women find new spiritual sustenance and assistance, thus reducing the impact of stigma on infertile women FertiQoL. This is consistent with previous research results on meaning-based coping being conducive to the improvement of marital relationship and FertiQoL in infertile women [26, 32].

In addition to the mediating effects of the four coping strategies, our results also indicated that stigma may play an important role as an internal resource mediated by coping strategies in managing and controlling daily life among infertile women. Goffman claimed that no matter whether the stigma is visible or hidden, an individual may experience discrimination [33]. This means that the stigmatization that infertile women experience is deeply rooted in her perceived discrepancy between what society dictates of her and the factual standard of her identity. This has been linked with an array of negative consequences – the level of stigma is positively related to negative emotions such as stress and anxiety, where high levels of stigma affects the behavior of infertile women seeking medical treatment, thus leading to a further deterioration in FertiQoL [13, 16, 34]. As the results of this study showed, there was a significantly negative correlation between stigma and FertiQoL. This is also supported by previous qualitative studies, where infertile women perceived that their daily lives were disrupted, their marital relationship to have deteriorated, and complained that they had lower social status and social support during fertility treatment [27, 35].

### Strengths and limitations

In this study, we focus on infertile women in a special period, who are undergoing IVF-ET treatment which is regarded as the last infertile straw for infertile women and has a significant impact on FertiQoL of these women. Therefore, in this study, we also we pay more attention to the FertiQoL of these women, and find the impact path on stigma and coping strategies on their FertiQoL, which not only provided evidence for improving the level of FertiQoL on infertile women, but also provided a direction psychological intervention during the treatment of infertility.

Convenience sampling was used in this study and the participants were recruited from a single center, which may have caused sampling bias and limited the generalizability of the findings to the population of women with infertility in China. Additionally, the male counterparts and existing social support of the participants in this study was not investigated, limiting study findings. Furthermore, as the clinical pregnancy outcomes of the participants were not traced in this cross-sectional study, the causal relationship between variables studied cannot be established.

## Conclusion

Our study showed a significant association between stigma and FertiQoL, with a mediating role of coping strategy. Avoidance coping strategy was identified to play the most important negatively mediating factor between stigma and FertiQoL in Chinese infertile women undergoing IVF-ET treatment. In addition, meaning-based coping strategy played a significantly positive mediating role. Finding from this study can guide public healthcare campaigns in addressing both self-stigma of infertile women and the social stigma that these women encounter at home with their families and in the wider community.

## Data Availability

The data used and/or analyzed during the current study are available from the corresponding author on reasonable request.

## Abbreviations

FertiQoL: Fertility quality of life
IVF-ET: In vitro fertilization embryo transfer
ISS: Infertility Stigma Scale
